# Human platelet lysate-cultured adipose-derived stem cell sheets promote angiogenesis and accelerate wound healing via CCL5 modulation

**DOI:** 10.1186/s13287-024-03762-9

**Published:** 2024-06-09

**Authors:** Yueh-Chen Chen, Er-Yuan Chuang, Yuan-Kun Tu, Chia-Lang Hsu, Nai-Chen Cheng

**Affiliations:** 1 Department of Surgery, National Taiwan University Hospital and College of Medicine, 7 Chung-Shan S. Rd, Taipei, 100 Taiwan; 2 International Ph.D. Program in Biomedical Engineering, Graduate Institute of Biomedical Materials and Tissue Engineering, Taipei Medical University, Taipei, Taiwan; 3 Department of Orthopedics, E-Da Hospital/I-Shou University, Kaohsiung, Taiwan; 4 Department of Medical Research, National Taiwan University Hospital, Taipei, Taiwan; 5 Research Center for Developmental Biology and Regenerative Medicine, National Taiwan University, Taipei, Taiwan

**Keywords:** Adipose-derived stem cells, Cell sheet, Wound healing, Angiogenesis, CCL5

## Abstract

**Background:**

A rising population faces challenges with healing-impaired cutaneous wounds, often leading to physical disabilities. Adipose-derived stem cells (ASCs), specifically in the cell sheet format, have emerged as a promising remedy for impaired wound healing. Human platelet lysate (HPL) provides an attractive alternative to fetal bovine serum (FBS) for culturing clinical-grade ASCs. However, the potential of HPL sheets in promoting wound healing has not been fully investigated. This study aimed to explore the anti-fibrotic and pro-angiogenic capabilities of HPL-cultured ASC sheets and delve into the molecular mechanism.

**Methods:**

A rat burn model was utilized to evaluate the efficacy of HPL-cultured ASC sheets in promoting wound healing. ASC sheets were fabricated with HPL, and those with FBS were included for comparison. Various analyses were conducted to assess the impact of HPL sheets on wound healing. Histological examination of wound tissues provided insights into aspects such as wound closure, collagen deposition, and overall tissue regeneration. Immunofluorescence was employed to assess the presence and distribution of transplanted ASCs after treatment. Further in vitro studies were conducted to decipher the specific factors in HPL sheets contributing to angiogenesis.

**Results:**

HPL-cultured ASC sheets significantly accelerated wound closure, fostering ample and organized collagen deposition in the neo-dermis. Significantly more retained ASCs were observed in wound tissues treated with HPL sheets compared to the FBS counterparts. Moreover, HPL sheets mitigated macrophage recruitment and decreased subsequent wound tissue fibrosis in vivo. Immunohistochemistry also indicated enhanced angiogenesis in the HPL sheet group. The in vitro analyses showed upregulation of *C–C motif chemokine ligand 5* (*CCL5)* and *angiogenin* in HPL sheets, including both gene expression and protein secretion. Culturing endothelial cells in the conditioned media compared to media supplemented with CCL5 or angiogenin suggested a correlation between CCL5 and the pro-angiogenic effect of HPL sheets. Additionally, through neutralizing antibody experiments, we further validated the crucial role of CCL5 in HPL sheet-mediated angiogenesis *in vitro.*

**Conclusions:**

The present study underscores CCL5 as an essential factor in the pro-angiogenic effect of HPL-cultured ASC sheets during the wound healing process. These findings highlight the potential of HPL-cultured ASC sheets as a promising therapeutic option for healing-impaired cutaneous wounds in clinical settings. Furthermore, the mechanism exploration yields valuable information for optimizing regenerative strategies with ASC products.

**Brief acknowledgment:**

This research was supported by the National Science and Technology Council, Taiwan (NSTC112-2321-B-002-018), National Taiwan University Hospital (111C-007), and E-Da Hospital-National Taiwan University Hospital Joint Research Program (111-EDN0001, 112-EDN0002).

**Supplementary Information:**

The online version contains supplementary material available at 10.1186/s13287-024-03762-9.

## Introduction

Burn injuries are prevalent in populations with lower socioeconomic status, but recent advances in burn care have contributed to reducing the incidence and severity [1]. Prolonged healing process in burn patients may lead to various complications, including wound infection and other morbidities. Burn injuries not only attenuate skin vitality in the wounded area but also damage surrounding vessels, impeding the supply of essential nutrients and oxygen. Increased vascular permeability can, in some cases, progressively lead to hypovolemic shock in patients with extensive burn injuries [2]. Therefore, timely and efficient management of burn wounds to restore adequate blood perfusion is imperative to enhance healing.

Adipose-derived stem cell (ASC) has emerged as a promising option of mesenchymal stem cells (MSCs) in regenerative medicine. ASCs are readily accessible and abundant in adipose tissues, constituting approximately 1% of total cells, a substantially higher number compared to the ratio of 0.001–0.002% found among those derived from bone marrow [3]. Despite their accessibility, a significant challenge in utilizing dissociated ASCs in clinical settings is their tendency to lose efficacy after transplantation. Consequently, the three-dimensional culture technology of ASCs has garnered considerable attention, showing robust therapeutic potential in many disease conditions [4]. Recently, cell sheets generated from multiple sources have been widely applied in varied diseases, including cartilage defects, cardiomyopathy, bone and nerve repair [5–8]. Cell sheets have been employed in wound healing because of their capability to support the process of re-vascularization and re-epithelialization. Particularly, multi-layer ASC sheets presented as a promising treatment for impaired wound healing, including diabetic ulcers, limb ischemia, and oral ulcers [9–11].

Using temperature-responsive surfaces for culturing cell sheets has been developed for regenerative therapies [12], but it requires specialized biomaterial and is relatively expensive. By supplementing _L_-ascorbate 2-phosphate (A2-P), we have successfully fabricated cell sheets consisting of multi-layered ASCs without the use of specialized biomaterials. Bountiful extracellular matrix (ECM) formation was deposited in these sheets, including a strong meshwork composed of collagen, fibronectin and laminin [9]. Moreover, ASCs residing in these cell sheets exhibited enhanced stemness markers, such as *NANOG*, *OCT4*, and *SOX2*, and several growth factors/cytokines, including hepatocyte growth factor (HGF). Cell sheets also enhanced healing in a murine wound model, primarily attributed to the secretion of certain paracrine factors [10]. Similarly, better prognosis was noted in the ischemic heart treated by cell sheets, as compared to the injection of the dissociated cells into the infarcted area [13]. Collectively, previous studies revealed higher regenerative and immunomodulatory potentials of ASC sheets. In comparison to the transplantation of dissociated cells, grafting cell sheets could diminish the unpredictable loss of cells, thus enhancing the therapeutic efficacy by retaining a great number of cells in the injured tissues.

The transplanted ASC sheets have exerted beneficial effects on tissue healing in different experimental animal models, suggesting their potential application in the forthcoming translational studies [4]. To accomplish the generation of clinical-grade cell sheet products, human platelet lysate (HPL) was utilized as a substitute for xenogeneic fetal bovine serum (FBS) in the medium to circumvent biosafety issues and possible constraints [14, 15]. A meta-analysis demonstrated that HPL could accelerate the cell proliferation rate and decrease the doubling time of MSCs compared to the use of FBS in culture. Additionally, supplementation of HPL enriched the paracrine activities of MSCs while simultaneously maintaining their immunomodulatory abilities [16]. Chemokines released by stem cells can coordinate the trafficking of various cell types and mediators in the sequential phases of cutaneous wound healing [17]. Particularly, ASCs secrete angiogenic factors, thus supporting neovascularization in injured regions to foster wound healing [18], with CCL5 being one of them. The C–C motif chemokine ligand 5 (CCL5), also known as RANTES (regulated upon activation, normal T cell expressed and secreted), is an important member among the secretome of ASC. While CCL5 has been reported to recruit diverse residing and infiltrating cells, it can also participate directly in the process of angiogenesis [19]. Our research group previously reported that the conditioned medium of HPL sheets exhibited a higher angiogenic potential compared to the FBS counterpart in vitro and *in ovo.* However, the efficacy of HPL sheets in enhancing wound healing has not been thoroughly investigated in animal models. Therefore, we aimed to assess the regenerative properties of HPL sheets in vivo and further decipher the underlying mechanism of their pro-angiogenesis effects during wound healing.

## Materials and methods

### Isolation of human ASCs and cell culture

ASCs were isolated from the subcutaneous fat tissue of four non-diabetic, non-smoking female donors with an average age of 45 y (32–57 y) and an average body mass index of 24.6 (21.0–26.6) [20]. The study protocol has received approval from the Research Ethics Committee of National Taiwan University Hospital, and the informed consent was acquired from each adipose tissue donor participating in this study. This study was conducted in compliance with the institutional biosafety regulations. Small pieces of subcutaneous fat tissue were finely minced and subsequently rinsed with phosphate-buffered saline (PBS; Omics Biotechnology, Taipei, Taiwan), followed by enzymatic digestion using collagenase type I (Gibco, Carlsbad, CA, USA) for 1 h. After centrifuging the cell suspension, pellets were suspended and plated with Dulbecco’s modified Eagle’s medium (DMEM)/F-12 (Hyclone) which is supplemented with 10% fetal bovine serum (FBS; Hyclone, Logan, UT, USA), 1% penicillin–streptomycin (Biological Industries, Kibbutz Beit Haemek, Israel), and 1 ng/mL FGF2 (R&D systems, Minneapolis, MN, USA). The cells were cultured in a humidified atmosphere with 5% CO_2_ at 37 °C, and the medium was refreshed every 2–3 days. The cells were detached using 0.05% trypsin-EDTA (Biological Industries) upon reaching 90% confluence and re-plated until the third or fourth passage for various experiments. These cells have been previously tested to exhibit differentiation capabilities toward multi-lineages [21, 22]. Isolated human ASCs were pooled into a single population due to their similar surface marker expression and comparable differentiation potential, as exhibited by each ASC clone.

### Fabrication of ASC sheets

ASC sheets were prepared and characterized as described previously [23]. Briefly, ASCs were seeded at a density of 1 × 10^4^ cells/cm^2^ in a 0.1% gelatin coated culture dish or plate. The culture medium consisted of DMEM-high glucose (DMEM-HG; Hyclone), 1% penicillin/ streptomycin (Biological Industries), 0.02% heparin (Sigma-Aldrich, St. Louis, MO, USA), 250 µM _L_-ascorbate 2-phosphate (A2-P; Sigma-Aldrich), and supplemented with 10% FBS or 5% HPL (UltraGRO™; HELIOS BioScience, Atlanta, GA). The culture medium was refreshed every 2 ~ 3 days. As for the harvest of conditioned medium, the cultured medium of cell sheet was switched to DMEM-HG for additional 2 days.

### In vivo burn wound model

Male Wistar rat, aged 10–12 weeks, were purchased from BioLASCO Taiwan (Taipei, Taiwan). All experiments were performed in compliance with Guidance for Care and Use of Laboratory Animals and approved by the Taipei Medical University Administrative Committee on Animal Research (LAC-2018-0130) and ARRIVE (Animal Research: Reporting of In Vivo Experiments) 2.0 guidelines. All experiment procedures were conducted under general anesthesia (isoflurane, 3–5%, 0.5 L/min). After the hair at the rat dorsum was shaved, a stainless-steel cylinder (1 cm in diameter) was heated to 100 °C in a boiled water bath for 8 min, and then placed onto the back of rats for 15 s to induce partial-thickness burn wounds [24]. Four 1 cm-diameter round burn wounds were created on the back of each rat, and the wounds were immediately photographed using a digital camera (Ricoh, Ohta-ku, Tokyo, Japan). In each rat, one wound was selected for PBS injection as controls, and the other three wounds were treated with HPL-cultured or FBS-cultured ASC sheets respectively. Subsequently, the dorsal skin was covered with a transparent, semi-occlusive adhesive dressing (Tegaderm; 3 M, St. Paul, MN, USA) for wound protection. The wounds were observed and photographed on post-injury day 0, 7, 14, 21 and 28. Each wound was evaluated by four blinded investigators on a rating from 0 to 4 based on (1) brown discoloration and (2) scabbing/hardness (Additional file1: Table S1) [24]. The wound images were analyzed using ImageJ software to measure wound areas, with the wound size on day 0 defined as 100%. All animals were euthanized by carbon dioxide inhalation and the entire wound tissues were harvested on post-injury day 5 and day 28 for further analysis (*n* = 3 for each time point in each group).

### Histological analysis and immunohistochemistry

Wound tissue samples were snap frozen in liquid nitrogen for frozen sections or fixed in 4% paraformaldehyde overnight and dehydrated before paraffin embedding. Sections were made perpendicular to both the wound surface and the anterior-posterior axis, cutting into 4 μm for histological analysis. In order to visualize the histological changes and collagen deposition, Hematoxylin and Eosin (H&E) and Masson’s trichrome staining were employed to stain the sections according to the manufacturer’s protocol (Sigma-Aldrich). Three sections per group were randomly chosen, and five high-power fields (hpfs) in each section were randomly selected and measured for skin thickness and collagen density.

For immunohistochemical staining, rat wound tissue sections were deparaffinized in xylene and rehydrated in a series concentration of ethanol. The antigen was unmasked using pH 9.0 Tris-EDTA buffer (Abcam, Cambridge, UK) at 95 °C for 30 min. Tissue sections were blocked with 3% bovine serum albumin (BSA; BioShop, Burlington, Ontario, Canada) before the incubation with primary antibodies. Then the sections were labeled with the primary antibodies against CD31 (Abcam), CD68 (Abcam), α-smooth muscle actin (α-SMA; Abcam), and transforming growth factor-β1 (TGF-β1; Santa Cruz Biotechnology, Dallas, Texas, USA) at 4 °C overnight. The secondary antibody linking to horseradish peroxidase (R&D systems) was incubated for 1 h at room temperature. All specimens were visualized by a 3,3’-Diaminobenzidine chromogen kit (BIOTnA Biotech, Kaohsiung, Taiwan), and the nuclei were counterstained with hematoxylin solution (Abcam). The stained sections were scanned by TissueFAXS scanning system (TissueGnostics, Vienna, Austria) and analyzed using StrataQuest software. The signals were quantified in 5 randomly selected high-power fields per section from three different sections.

### Immunofluorescence staining

The paraffin-embedded tissues were cut into 4-µm sections, mounted on slides, deparaffinized, dehydrated, and the antigen was retrieved by pH 9.0 Tris-EDTA buffer at 70°C for 20 min. The sections were incubated in 3% BSA with 0.1% triton (Sigma-Aldrich) for blocking non-specific protein binding. The staining of transplanted human ASCs was conducted using anti-human nuclear antigen (HNA; Merck Millipore, Darmstadt, Germany) overnight at 4°C, followed by incubation with a fluorescence-conjugated secondary antibody (Alexa Fluor 594-conjugated goat anti-mouse IgG; BioLegend, San Diego, CA, USA) at room temperature for 1 h. Nuclei were counterstained with 4’, 6-diamidino-2-phenylindole (DAPI; Santa Cruz Biotechnology). To rule out non-specific labeling, negative controls lacking primary antibodies were also employed. The sections were analyzed using a fluorescent microscope (EVOS™ M7000 Imaging System; Invitrogen, Waltham, MA, USA), and the immunolabeled cells were quantified in 3 randomly selected high-power fields per section from three different sections.

### Microarray processing and gene expression analysis

Total RNA from FBS-cultured or HPL-cultured ASC sheets was prepared using TRIzol™ Reagent (Invitrogen). Residual genomic DNA was removed by DNase I digestion. RNA quantification was performed using a NanoDrop-1000 spectrophotometer (Thermo Fisher Scientific, Wilmington, DE, USA), and its quality was assessed through agarose electrophoresis and Bioanalyzer 2100 with an RNA 6000 Nano Kit (Agilent Technologies, Santa Clara, CA, USA). RNA expression in HPL and FBS-cultured ASC sheets was analyzed using SurePrint G3 Human Gene Expression 8 × 60 K Microarray (Agilent Technologies). RNA was amplified and labeled using Low Input Quick-Amp Labeling kit (Agilent Technologies) following the manufacturer’s instructions. Then, the correspondingly fragmented labeled RNA was pooled and hybridized to Agilent SurePrint Microarray. The chip was scanned with an Agilent microarray scanner and analyzed by Feature extraction 10.5.1.1 software (Agilent Technologies). The raw microarray data were submitted to NCBI Gene Expression Omnibus (GEO) repository with accession number GSE252798. For gene expression analysis, the microarray raw signals underwent normalization using the quantile method. Differential expression analysis was conducted using the NOISeq R package [25]. Genes with a probability of differential expression equal to or greater than 0.8 were considered as differentially expressed genes. Pre-rank gene-set enrichment analysis (GSEA) was performed utilizing the functions of the R package clusterProfiler [26]. Gene-sets from MSigDB (v7.4) were employed for this analysis [27]. Genes were ranked based on the product of their rank and probability values, obtained from the NOISeq results. This approach enhances the sensitivity of GSEA by incorporating both the degree of differential expression and the statistical confidence in the ranking.

### Angiogenesis array

Analysis of secretion profile in the conditioned medium of FBS or HPL-cultured ASC sheets was performed using a Human Angiogenesis Array C1 (RayBiotech, Norcross, GA, USA) according to the manufacturer’s instructions, allowing semi-quantitative determination of protein levels of a variety of pro-angiogenic factors.

### RNA isolation and quantitative polymerase chain reaction

Total RNA was extracted from FBS or HPL-cultured ASC sheets utilizing a RNeasy Mini Kit (Qiagen, Valencia, CA, USA) in accordance with the manufacturer’s protocols. Total RNA concentration was determined by optical density at 260 nm (OD_260_) using a spectrophotometer (Tecan, Männedorf, Switzerland) and reverse-transcribed into complementary DNA (cDNA) using High-Capacity cDNA Reverse Transcription Kits (Applied Biosystems, Foster City, CA, USA). Quantitative PCR was performed using a FastStart Universal SYBR Green Master (Roche, Indianapolis, IN, USA) and CFX Connect Real-Time PCR Detection System (Bio-Rad, Hercules, CA, USA). The gene expression level was normalized to glyceraldehyde 3-phosphate dehydrogenase (GAPDH) for each cDNA sample. A comparative threshold cycle method (ΔΔCt) was adopted to analyze the relative quantity (RQ) of mRNA expression between treated and corresponding control samples. The sequences of the used primers are shown in Additional file1: Table S2.

### Enzyme-linked immunosorbent assay (ELISA)

The quantitative analysis of C–C motif chemokine ligand 5 (CCL5) and angiogenin in the conditioned medium from FBS or HPL-cultured ASC sheets was performed using ELISA (Quantikine; R&D Systems). Optical densities were determined using a spectrophotometer (Tecan, Männedorf, Switzerland) at 450 nm, with wavelength correction set to 570 nm. Values were presented as the concentration of secreted CCL5 or angiogenin per 10^5^ cells at the time of harvest.

### In vitro proliferation assay

Human umbilical vein endothelial cells (HUVECs; PromoCell, Heidelberg, Germany) were seeded in a 0.1% gelatin-coated 96 well plate with serum supplemented endothelial cell growth medium 2 (EGM2; PromoCell). After 24 h-cell attachment, the cells were treated by CCL5 (R&D systems; 1 ng/mL) or angiogenin (R&D systems; 1, 10 and 50 ng/mL) in a serum-free medium composed of endothelial basal medium (EBM; PromoCell) and DMEM-HG (EBM/DMEM, 1:1). The conditioned medium from the HPL sheet was mixed with equal volume of EBM. The HUVECs were incubated for additional 24 h. The aforementioned basal medium (EBM/DMEM) served as a negative control. The proliferation was assessed by Alamar Blue assay (AbD Serotec, Kidlington, United Kingdom). The fluorescence signals were measured at an excitation wavelength at 560 nm and an emission wavelength at 590 nm by a spectrometer (Tecan). The activity index of ASCs was defined as the proliferative rate of each group relative to the control.

### In vitro tube formation assay

The capability of HUVECs to form capillary-like structures was further evaluated in the tube formation assay as described previously [23]. In brief, HUVECs were seeded on pre-chilled µ-slides (Ibidi, Grafelfing, Germany) coated with growth factor reduced Matrigel basement membrane matrix (Corning, Lowell, MA, USA) at a density of 7,500 cells/well. HUVECs were treated by CCL5 (1, 10, 50 ng/mL) or angiogenin (1, 10, 50 ng/mL) in the aforementioned EBM/DMEM medium. The conditioned medium from FBS sheet or HPL sheet was mixed with equal volume of EBM before treatment. The neutralizing CCL5 antibody (R&D systems; 75 ng/mL) was applied in the HPL sheet derived conditioned medium to examine the contribution of CCL5. The aforementioned basal medium (EBM/DMEM) served as a negative control, while serum supplemented EGM2 was used as a positive control. Formation of tube-like structures was visualized by a phase-contrast microscope after 6 h incubation. The images were analyzed using ImageJ software as described previously [28].

### Statistical analysis

All measurements are reported as means ± standard deviation. Statistical significance was evaluated using one-way analysis of variance (ANOVA) followed by Tukey’s multiple comparison tests or Student’s t test to compare the results of control and the experimental groups. All experimental data were analyzed using GraphPad Prism 8 (GraphPad Software, Boston, MA, USA). Statistical significance was defined as *p* < 0.05.

## Results

### Wound healing ability of HPL-cultured sheets in a rat model

**Fig. 1:**
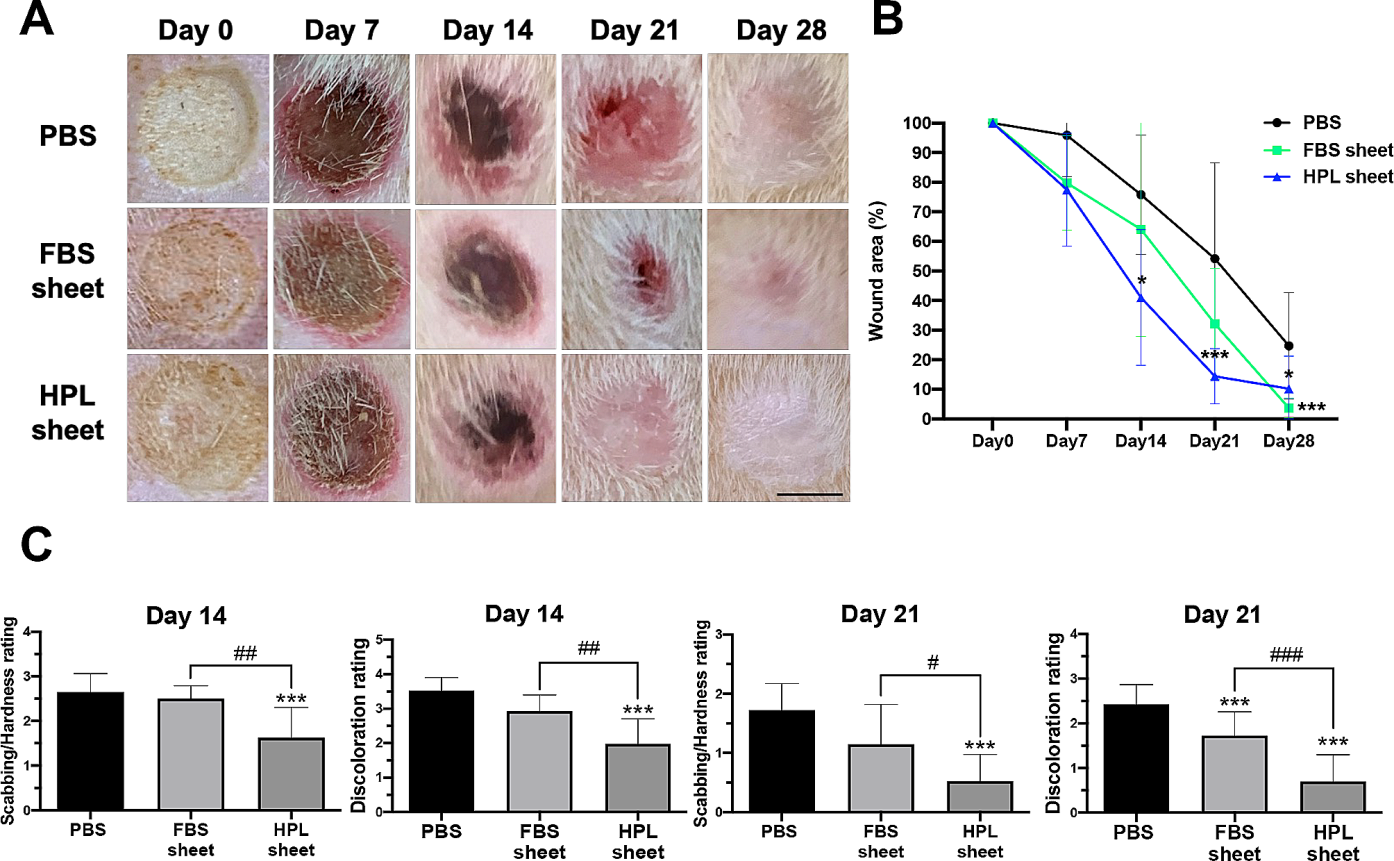
ASC sheets accelerated burn wound healing in a murine model. (**A**) Gross pictures of burn wounds on post-injury day 0, 7, 14, 21 and 28. Scale bar = 5 mm. (**B**) Wound closure curves of the PBS, FBS sheet, and HPL sheet group. (**C**) Quantification of wound healing index: discoloration and scabbing/hardness as examination for effectiveness of wound repair. *p < 0.05, ***p < 0.001 from PBS control, ^#^*p* < 0.05; ^##^*p* < 0.01; ^###^*p* < 0.001 between the indicated groups, ANOVA followed by Tukey’s multiple comparison test. Values are presented as means ± SD. ASC, adipose-derived stem cell; FBS, fetal bovine serum; HPL, human platelet lysate

The in vivo wound healing effect of ASC sheets was evaluated in a rat burn model on post-injury day 0, 7, 14, 21 and 28 (Fig. 1A). A significantly smaller wound area was noticed only in the HPL sheet group on post-injury day 14 and 21 relative to the PBS group (Day 14: 41.0% ± 22.9% vs. 75.8% ± 20.2%, *p < 0.05; Day 21: 14.4% ± 9.3% vs. 54.2% ± 32.4%, ***p < 0.001; Fig. 1B). On day 28, the wounds treated by FBS sheets or HPL sheets were significantly smaller than the control (FBS sheet: 3.7% ± 3.4%; HPL sheet: 10.2% ± 11.1% vs. PBS: 24.7% ± 18.0%; ***p < 0.001 and *p < 0.05 respectively).

A wound healing index of brown discoloration and scabbing/hardness was employed to evaluate the effectiveness of the treatment for burn wounds. The healing process of the burn wounds exhibited a progressive transition from a dark brown hue to the formation of scabs, ultimately returning to a healthy pink shade. On day 14, the wounds treated with HPL sheets showed significantly lower discoloration rating (HPL sheet: 2.0 ± 0.7; ***p < 0.001 vs. PBS: 3.5 ± 0.4) and a significantly lower scabbing/hardness rating was noted in HPL sheet group on day 21 (0.5 ± 0.5 vs. 1.7 ± 0.4, ***p < 0.001; Fig. 1C). These ratings were in line with the wound healing trends among different groups.

### Histological examination of wound tissues

**Fig. 2:**
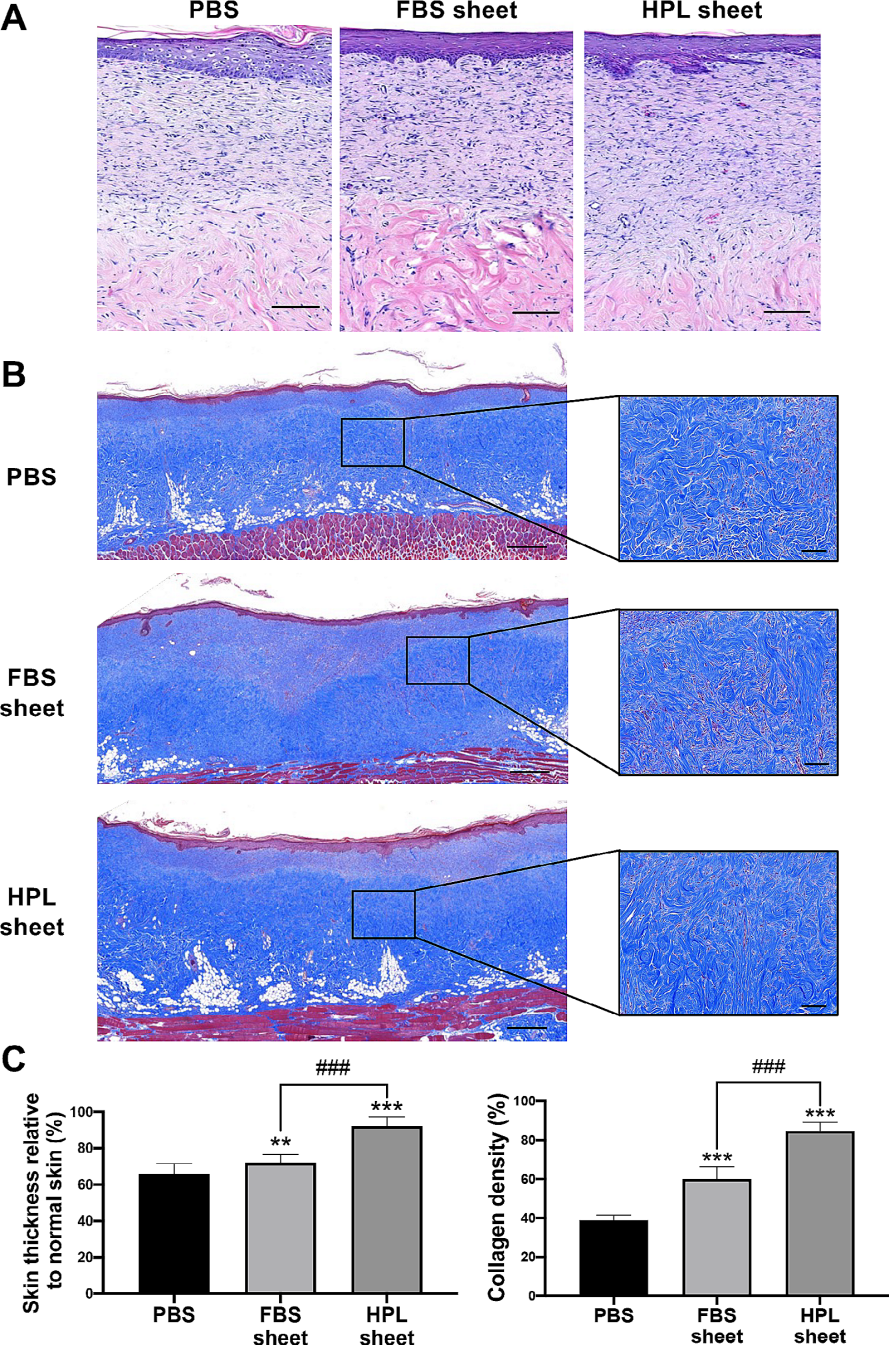
Histology of healed wound tissues in rats undergoing PBS, FBS sheet and HPL sheet treatment on post-injury day 28. (**A**) Representative images of H&E staining. Scale bar = 100 μm. (**B**) Representative images of Masson’s trichrome staining. Scale bar = 500 μm; higher magnification scale bar = 100 μm. (**C**) Collagen densities of neo-dermis in wound tissues were calculated (*n* = 5) and also the quantification of relative skin thickness ratio of the wounded sites and normal rat skin of wound tissues (*n* = 5). Image analysis performed on 5 random fields per tissue section and 3 sections per condition; **p < 0.01, ***p < 0.001 from PBS control; ^###^*p* < 0.001 between the indicated groups, ANOVA followed by Tukey’s multiple comparison test. Values are presented as means ± SD. FBS, fetal bovine serum; HPL, human platelet lysate; Η&Ε, hematoxylin and eosin

The histological morphology of wound tissues on day 28 were evaluated by H&E staining (Fig. 2A), while Masson’s trichome staining was used to detect the collagenous tissue formation (Fig. 2B). The thickness of the neo-skin relative to the nearby normal skin was quantified. On day 28, the relative thicknesses in ASC sheet groups were significantly thicker than the PBS group (FBS sheet: 72.1% ± 4.4%, **p < 0.01; HPL sheet: 92.2% ± 5.2%, ***p < 0.001 vs. PBS: 65.7% ± 5.8%; Fig. 2C, left). The collagen density was also found to be significantly higher with more complex collagen structure in the neo-dermis of both ASC sheet treatment groups (FBS sheet: 60.1% ± 6.3% and HPL sheet: 84.7% ± 4.4% vs. PBS: 38.9% ± 2.6%, ***p < 0.001 respectively; Fig. 2C, right). Moreover, significantly more collagen deposition was noted in the HPL sheet treatment group relative to the FBS counterpart (84.7% ± 4.4% vs. 60.1% ± 6.3%, ^###^*p* < 0.001; Fig. 2C, right).

### HPL sheets facilitated angiogenesis in the wound tissues

**Fig. 3:**
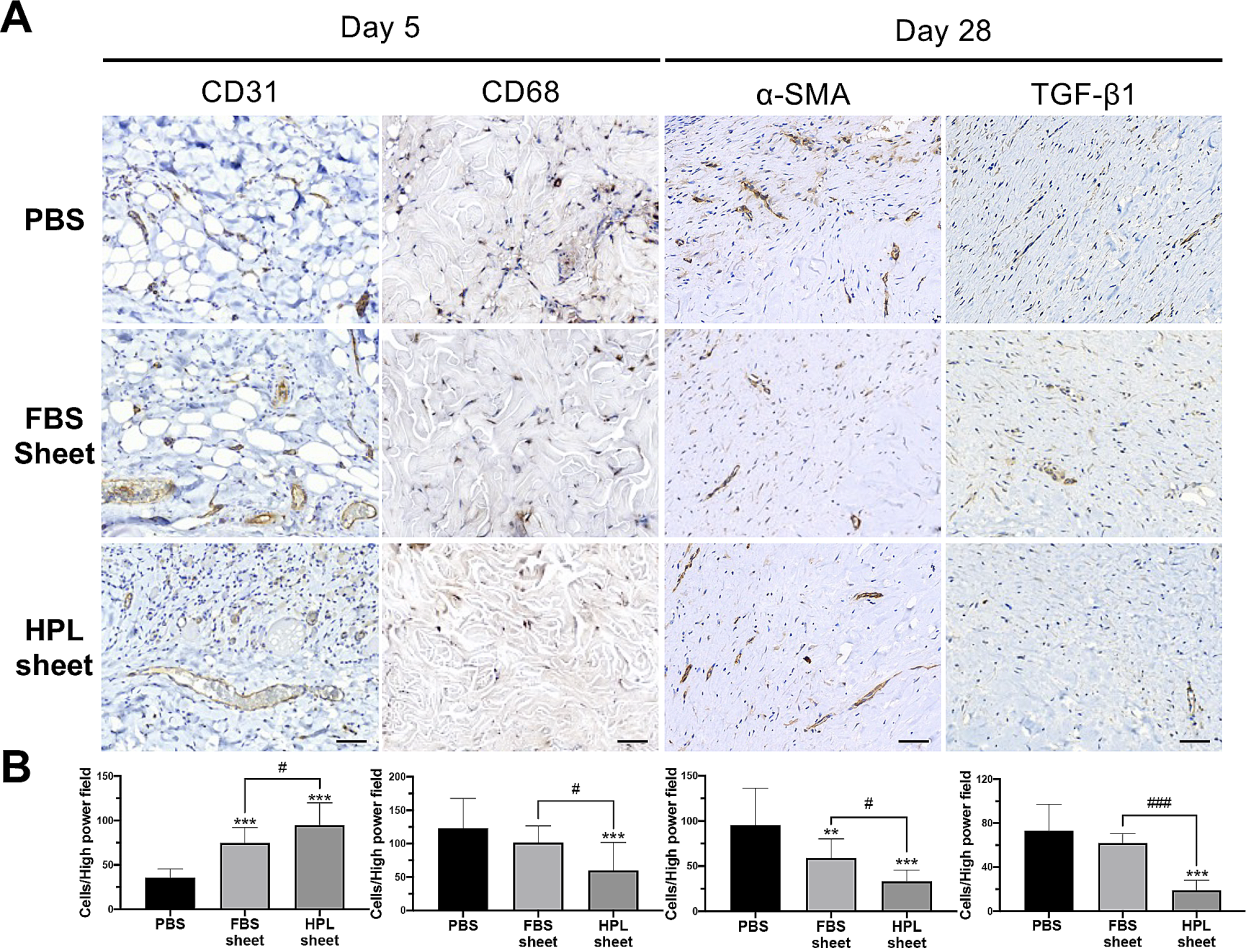
HPL sheet facilitated angiogenesis, suppressed macrophage infiltration, and eliminated the anti-fibrotic expression of α-smooth muscle actin (α-SMA) and transforming growth factor-β1 (TGF-β1) in burn wound tissues. (**A**) Representative images of immunohistochemical sections on day 5 and day 28 from different groups. Scale bar = 50 μm. (**B**) Quantitative analysis of immunohistochemical signals of CD31, CD68, α-SMA and TGF-β1 in high power field images. Image analysis performed on 5 random fields per tissue section and 3 sections per condition; **p < 0.01, ***p < 0.001 from PBS control; ^#^*p* < 0.05, ^###^*p* < 0.001 between the indicated groups, ANOVA followed by Tukey’s multiple comparison test. Values are presented as means ± SD. FBS, fetal bovine serum; HPL, human platelet lysate; CD, cluster of differentiation

Immunohistochemistry of CD31, an angiogenic marker, was analyzed in wound tissues harvested on post-injury day 5. Significantly more CD31-positive cells were found in HPL or FBS sheet groups relative to the control (HPL sheet: 94.7 ± 25.2 cells/hpf and FBS sheet: 74.8 ± 17.3 cells/hpf vs. PBS: 35.7 ± 9.8, ***p < 0.001 respectively). Comparing to the FBS group, significantly more CD31 signals were also noted in the wound sections receiving HPL sheets (^#^*p* < 0.05; Fig. 3B).

### HPL sheets suppressed the macrophage infiltration and wound tissue fibrosis

The immunohistochemical staining of CD68, TGF-β1 and α-SMA were conducted in the wound sections (Fig. 3A). On day 5, the wounds receiving HPL sheets exhibited significantly fewer CD68 + macrophages relative to the control (60.1 ± 41.9 cells/hpf vs. 123.2 ± 44.5 cells/hpf, ***p < 0.001) or the FBS sheet group (60.1 ± 41.9 cells/hpf vs. 101.6 ± 25.2 cells/hpf, ^#^*p* < 0.05; Fig. 3B). On day 28, α-SMA exhibited significantly fewer signals in both ASC sheet groups relative to the control (FBS sheet: 59.1 ± 21.1 and HPL sheet: 33.1 ± 12.4 cells/hpf vs. PBS: 95.5 ± 40.7 cells/hpf, **p < 0.01 and ***p < 0.001 respectively). Comparing to the FBS group, significantly fewer α-SMA signals were also noted in the wound sections receiving HPL sheets (^#^*p* < 0.05). Moreover, the HPL sheet group expressed significantly fewer signals of TGF-β1 compared to the PBS group (19.1 ± 9.0 cells/hpf vs. 72.9 ± PBS: 24.1 cells/hpf, ***p < 0.001) or the FBS group (19.1 ± 9.0 cells/hpf vs. 62.0 ± 8.7 cells/hpf, ^###^*p* < 0.001; Fig. 3B).

### ASC retention in the wound tissues

**Fig. 4:**
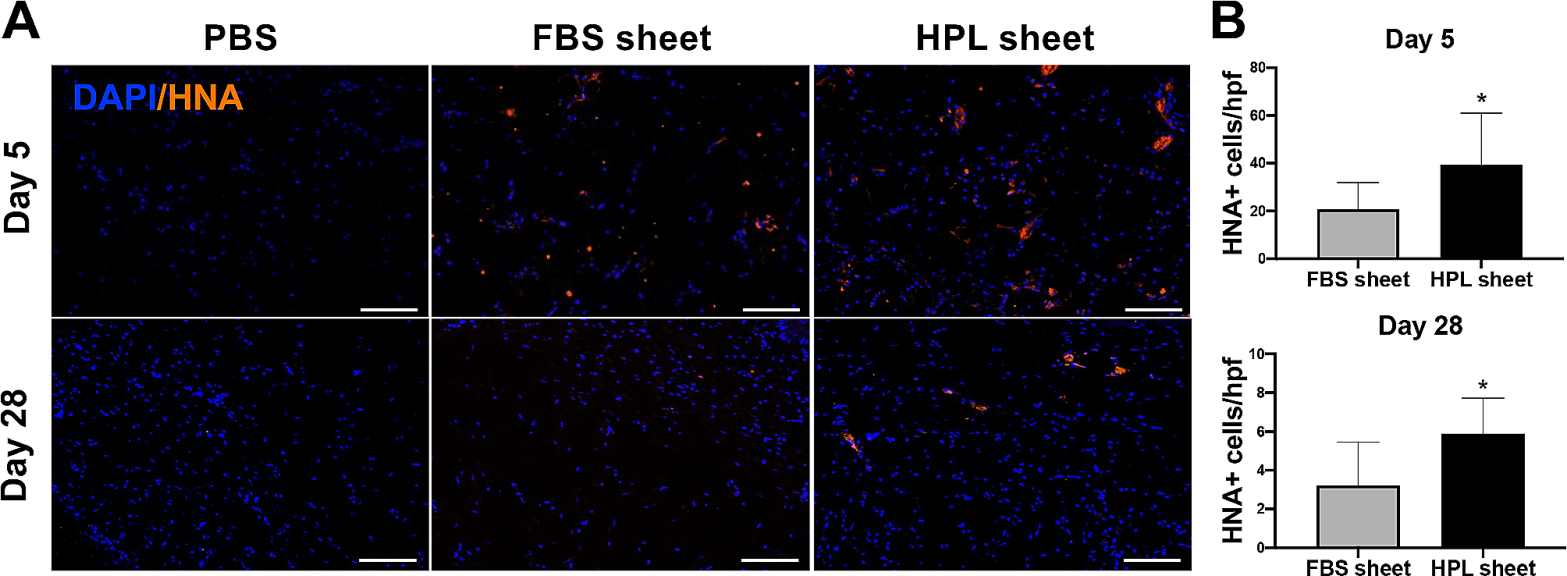
Retention of human ASCs in the wound tissues. (**A**) Immunofluorescent staining of HNA in the day 5 and 28 wound sections. Scale bar = 100 μm. (**B**) Quantification analysis of HNA-positive cells in the wound tissues on day 5 and day 28. Image analysis performed on 3 random high-power fields (hpf) per tissue section and 3 sections per condition, *p < 0.05 relative to the FBS sheet group, Student’s *t* test. Values are presented as means ± SD. FBS, fetal bovine serum; HPL, human platelet lysate; DAPI, 4’,6-diamidino-2-phenylindole; HNA, human nuclear antigen

To evaluate the retention of transplanted ASCs in the wound tissues, immunofluorescent staining with HNA was performed in the wound sections harvested on day 5 and 28 after sheet application (Fig. 4A). Significantly more HNA-positive cells were detected in the HPL sheet group compared to the FBS sheet group (39.2 ± 21.7 vs. 20.8 ± 11.1 cells/hpf, *p < 0.05) on day 5 and day 28 (5.9 ± 1.8 vs. 3.2 ± 2.2 cells/hpf, *p < 0.05; Fig. 4B). Meanwhile, the immunofluorescence signal of HNA was absent in wound tissues that received PBS injection.

### Angiogenic growth factor expression in HPL sheets

The gene expression pattern of HPL or FBS-cultured ASC sheets were analyzed using microarray (Additional file1: Figure S1A). Functional enrichment analysis showcased the top 10 hallmark gene-sets from MSigDB that were significantly enriched in either up-regulated or down-regulated genes. Notably, the angiogenesis-related pathway was upregulated in HPL sheets relative to the FBS counterpart (Additional file1: Figure S1B). The expression levels of angiogenesis-associated genes were further illustrated in a heatmap, depicting the different expression pattern between HPL and FBS sheets (Additional file1: Figure S1C).

**Fig. 5:**
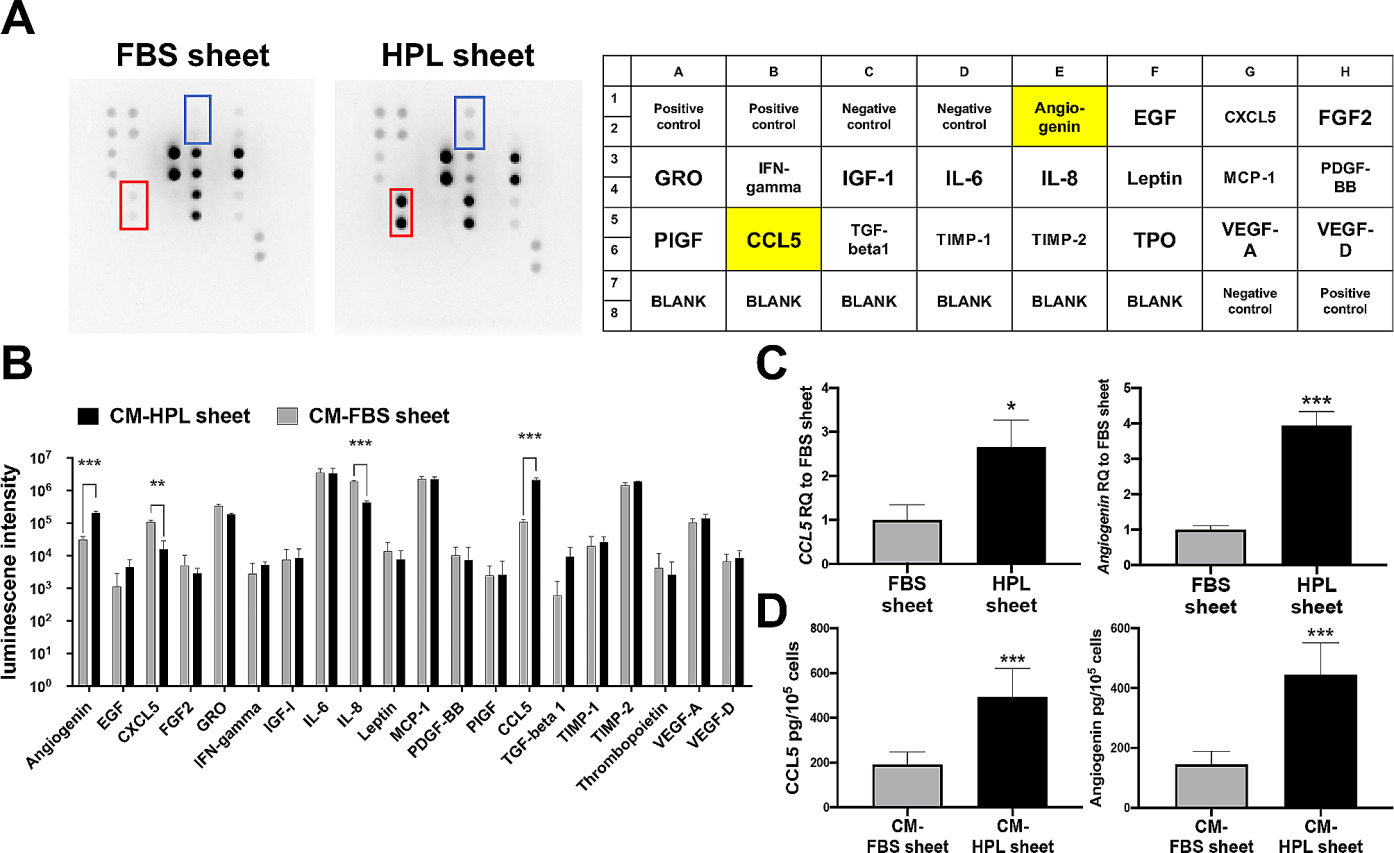
Enhanced expression of CCL5 and angiogenin in HPL sheets. (**A**) Angiogenesis-related proteins in the conditioned medium of FBS or HPL sheets were analyzed by Human Angiogenesis Array C1. Representative images of angiogenesis array and the indicated map were shown. Red boxes marked out CCL5 and blue boxes indicated angiogenin. (**B**) The secretion level of each protein was normalized by reference spots. The results revealed significantly enhanced expression of CCL5 and angiogenin in HPL sheet group (*n* = 3 samples/condition, **p < 0.01, ***p < 0.001 relative to the FBS group, multiple *t* test). (**C**) Quantitative PCR measurements for *CCL5* and *angiogenin* in FBS and HPL sheets (*n* = 3 samples/condition; *p < 0.05 and ***p < 0.001 relative to FBS sheet group, Student’s *t* test). (**D**) Release of CCL5 and angiogenin from FBS sheets and HPL sheets was determined by ELISA. Conditioned medium (CM) was from 3 independent batches (*n* = 9 samples/condition, ***p < 0.001 relative to the FBS sheet group, Student’s *t* test). Values are presented as means ± SD. FBS, fetal bovine serum; HPL, human platelet lysate; EGF, epidermal growth factor; CXCL5, C-X-C motif chemokine ligand 5; FGF2, basic fibroblast growth factor 2; GRO, growth-regulated oncogene; IFN-gamma, interferon-gamma; IGF-1, insulin growth factor-1; IL-6, interleukin-6; IL-8, interleukin-8; MCP-1, monocyte chemoattractant protein-1; PDGF-BB, platelet-derived growth factor subunit B; PlGF, placenta growth factor; CCL5, C-C motif chemokine ligand 5; TGF-beta1, transforming growth factor-beta 1; TIMP, tissue inhibitor matrix metalloproteinase; TPO, thrombopoietin; VEGF, vascular epidermal growth factor; CM, conditioned medium

An antibody array was conducted to estimate the release of 20 human angiogenesis related proteins from HPL and FBS-cultured ASC sheets (Fig. 5A). By semi-quantification, significantly higher content of CCL5 and angiogenin was detected in the conditioned medium of HPL sheets (CM-HPL sheet) relative to the FBS counterpart (CM-FBS sheet, ***p < 0.001 respectively; Fig. 5B). Subsequently, the relative mRNA expression of CCL5 and angiogenin in ASC sheets was analyzed by quantitative PCR. Compared to FBS sheets, the HPL group exhibited significant upregulation of *CCL5* (2.7 ± 0.6 -fold, *p < 0.05) and *angiogenin* (3.9 ± 0.4 -fold, ***p < 0.001, Fig. 5C). Further ELISA also revealed higher concentrations of CCL5 and angiogenin released from HPL sheets into conditioned medium relative to FBS sheets (CCL5: 493.8 ± 126.6 pg/mL/10^5^ cells vs. 191.5 ± 56.5 pg/mL/10^5^ cells, ***p < 0.001; angiogenin: 444.9 ± 106.6 pg/mL/10^5^ cells vs. 144.9 ± 44.2 pg/mL/10^5^ cells, ***p < 0.001; Fig. 5D).

### Effect of angiogenin and CCL5 on endothelial cells

**Fig. 6:**
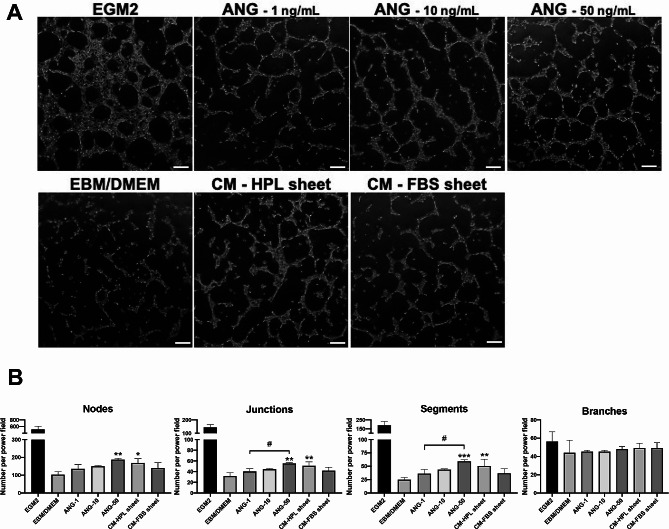
Effect of angiogenin on in vitro tube formation of human umbilical vein endothelial cells (HUVECs). (**A**) Representative microscopic images of HUVECs after 6 h-incubation in the presence of 1, 10, 50 ng/mL angiogenin. HUVECs cultured in endothelial cell growth medium 2 (EGM2) served as a positive control, and those in EBM/DMEM was a negative control. Scale bar = 200 μm. (**B**) Number of branching nodes, junctions, segments and branches per power field were compared among different groups. Tube formation of HUVECs cultured in low and medium level of angiogenin (1 ng/mL and 10 ng/mL) were not significantly higher than that observed in control condition. (*n* = 3; *p < 0.05, **p < 0.01 from control; ^#^*p* < 0.05 between the indicated groups, ANOVA followed by Tukey’s multiple comparison test). Values are presented as means ± SD. EBM/DMEM, endothelial cell growth basal medium/Dulbecco’s modified Eagle medium; ANG, angiogenin; FBS, fetal bovine serum; HPL, human platelet lysate; CM, conditioned medium

We assessed the influence of angiogenin and CCL5 on endothelial cells using in vitro tube formation assay. Several concentrations (1, 10, 50 ng/mL) of angiogenin were applied for HUVEC culture. After 6 h, the tube formation of HUVECs was quantified by counting the numbers of branching nodes, junction, segments and branches per power field (Fig. 6A). We observed a statistically significant increase in the numbers of nodes, junctions and segments only when the concentration of angiogenin was escalated to 50 ng/mL (***p < 0.001, **p < 0.01, *p < 0.05 relative to EBM/DMEM group; ^#^*p* < 0.05, between the indicated groups; Fig. 6B).

**Fig. 7:**
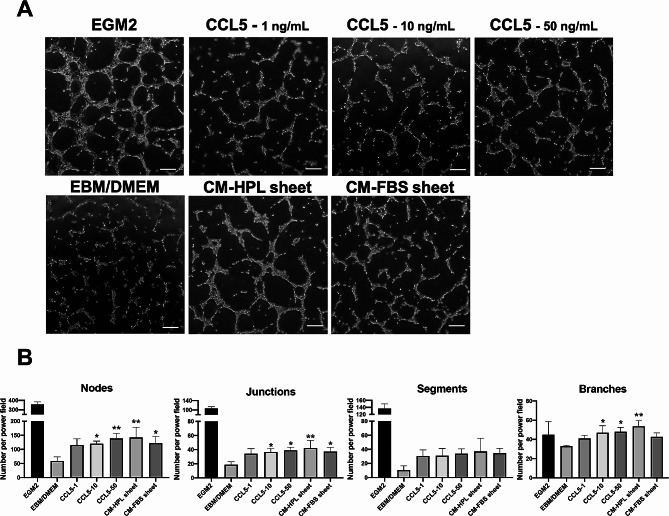
Effect of CCL5 on in vitro tube formation of human umbilical vein endothelial cells (HUVECs). (**A**) Representative microscopic images of HUVECs after incubation for 6 h in the presence of 1, 10, 50 ng/ml CCL5. HUVECs cultured in endothelial cell growth medium 2 (EGM2) served as a positive control, and those in EBM/DMEM was a negative control. Scale bar = 200 μm. (**B**) Number of branching nodes, junctions, segments and branches per power field were compared among different groups. Tube formation in response to 10 and 50 ng/mL of CCL5 was significantly higher than that observed in control condition with a dose-dependent pattern. (*n* = 3; *p < 0.05, **p < 0.01 from control, ANOVA followed by Tukey’s multiple comparison test). Values are presented as means ± SD. EBM/DMEM, endothelial cell growth basal medium/Dulbecco’s modified Eagle medium; CCL5, C-C motif chemokine ligand 5; FBS, fetal bovine serum; HPL, human platelet lysate; CM, conditioned medium

Similarly, various concentrations (1, 10, 50 ng/mL) of CCL5 were added in the HUVEC culture medium for 6 h (Fig. 7A). The data showed that both 10 and 50 ng/mL of CCL5 facilitated the formation of nodes, junctions and branches (**p < 0.01, *p < 0.05, relative to EBM/DMEM group; Fig. 7B). Furthermore, only the relatively high angiogenin concentration (50 ng/mL) could enhance the proliferation of HUVECs significantly (***p < 0.001, relative to EBM/DMEM group; Figure S2), while the equivalent efficacy on proliferation between CCL5 (1 ng/mL) and conditioned medium from HPL sheets was observed (CCL5 group: 1.3 ± 0.1-fold and CM-HPL sheet group:1.5 ± 0.1-fold, *p < 0.05, **p < 0.01, respectively; Figure S2). These results suggested that CCL5 plays an important role in HPL sheet-induced angiogenesis, while angiogenin may not be a major factor in HPL sheet-associated angiogenesis promotion.

### CCL5 neutralization inhibited tube formation of endothelial cells

**Fig. 8:**
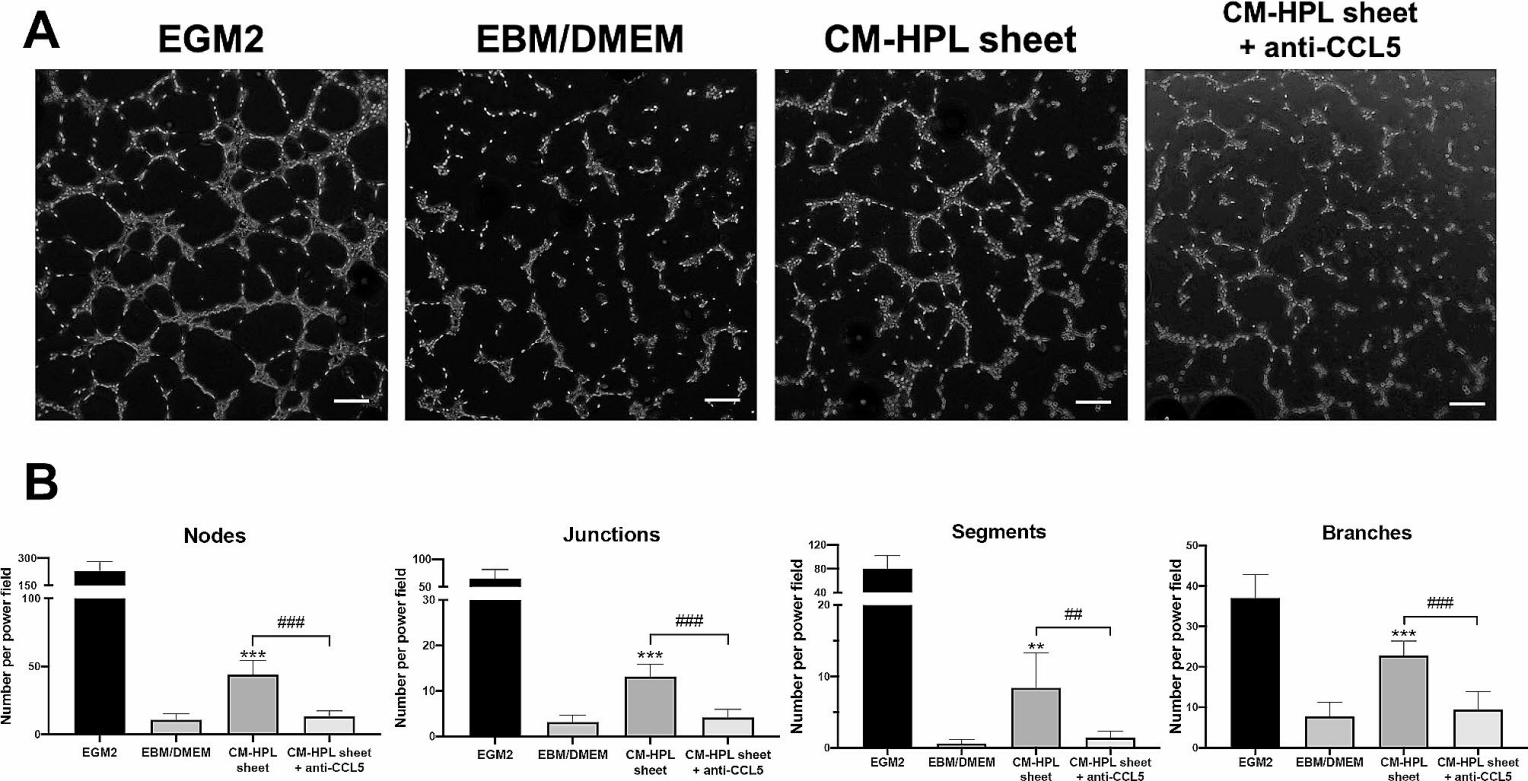
CCL5 neutralization in CM from HPL sheet inhibited angiogenesis of HUVECs. (**A**) Representative microscopic images of endothelial cells after incubation with CM from HPL sheets and CCL5-specific neutralizing antibody. HUVECs cultured in EGM2 served as a positive control, and those in EBM/DMEM was a negative control. Scale bar = 200 μm. (**B**) Number of branching nodes, junctions, segments and branches per power field were compared among different groups (*n* = 5; **p < 0.01, ***p < 0.001 from control; ^##^*p* < 0.01, ^###^*p* < 0. 001 between the indicated groups, ANOVA followed by Tukey’s multiple comparison test). Values are presented as means ± SD. EGM2, endothelial cell growth medium 2; EBM/DMEM, endothelial cell growth basal medium/Dulbecco’s modified Eagle medium; CCL5, C-C motif chemokine ligand 5; FBS, fetal bovine serum; HPL, human platelet lysate; CM, conditioned medium

To further validate the role of CCL5 in mediating the pro-angiogenic effect of HPL sheets, CCL5 neutralizing antibody was used to check whether the proangiogenic effects of CM-HPL sheet could be abolished. According to the concentration of CCL5 detected in the conditioned medium of HPL sheets and the neutralization curve of CCL5 antibody supplied by the manufacturer, we designed the neutralizing experiments by adding 75 ng/mL CCL5 neutralizing antibody. Neutralizing CCL5 inhibited in vitro tube formation in HUVECs induced by CM-HPL sheet (Fig. 8A). Relative to the CM-HPL sheet group, the group with additional CCL5 neutralizing antibody exhibited significantly decreased tube-like structures (13.2 ± 4.2 vs. 44.0 ± 10.3 for nodes; 4.2 ± 1.8 vs.13.2 ± 2.7 for junctions;1.4 ± 0.89 vs. 8.4 ± 4.9 for segments; 9.4 ± 4.5 vs. 22.8 ± 3.6 for branches, ^##^*p* < 0.01, ^###^*p* < 0.001, respectively; Fig. 8B). The results indicated that CCL5 is a key paracrine factor contributing to the pro-angiogenic potential of HPL sheets.

## Discussion

The wound healing process is orchestrated by a series of restorative reactions, including homeostasis, inflammation, proliferation and remodeling [29]. Burn wounds, if not healed in a timely fashion, may result in chronic healing-impaired wounds and serious complications. In the present study, HPL-cultured ASC sheets was found to be an effective treatment for burn injuries in a rat model, as evidenced by faster healing and lower ratings of brown discoloration and scabbing/hardness of the wound. Moreover, the neo-dermis in the HPL sheet treatment group exhibited a significantly higher collagen density, indicating abundant deposition of ECM molecules. Consistent with the previous demonstrated anti-fibrotic properties of ASC sheets in vitro [10], decreased levels of fibrosis-associated markers, α-SMA and TGF-β1, were observed in the wound tissues on day 28. The observation could be attributed to significantly fewer macrophage infiltrations into the wound tissues on post-injury day 5. Moreover, we found a higher retention of ASCs in wound tissues after the application of HPL sheets on day 5 and 28, potentially contributing to their superior therapeutic effects. Additionally, significantly more CD31 signals were detected in the day 5 wound tissues treated with HPL sheets, implying an enhanced pro-angiogenic effect relative to FBS sheets. Further in vitro investigation revealed a critical contribution of CCL5 released from HPL sheets in promoting angiogenesis, thus expediting the wound healing process.

Reported in a myriad of animal models and clinical cases, the involvement of MSCs in wound treatment has brought beneficial effects in promoting healing [30]. Three-dimensional cell culture authentically mimics the intrinsic chemical, mechanical, and biological traits of cellular niche in vitro, thereby positioning an advantageous role in stem cell-based therapies [4]. Different from the conventional scaffold-based tissue engineering technology, cell sheets preserve the cell-cell interactions and ECM structures, ensuring tissue stability and mitigating the immune reactions that may arise during cell transplantation. Kim et al. reported the stability and successful engraftment of umbilical cord derived-MSC sheets in immune-deficient mice [31]. Similarly, the scaffold-free ASC sheets were successfully fabricated and demonstrated to promote wound healing in several studies [11, 32]. To effectively translate ASC sheets from bench to bedside, the use of HPL as a supplement for culturing cell sheets has been advocated [33, 34]. The influence of HPL on cellular characteristics in ASC sheets and their functionalities has been investigated in vitro [23]. In the present study, we further applied HPL sheets to the burn wounds in rats to demonstrate their therapeutic effectiveness in vivo.

During the inflammatory phase of the wound repair process, activated neutrophils and macrophages are recruited from damaged blood vessels into the wounds, accompanied by the rapid release of various cytokines and chemokines [29]. At this moment, excessive macrophage infiltration can lead to prolonged inflammation, extensive fibrosis, and impaired remodeling [35]. In the present study, significantly fewer CD68 signals were observed in the day 5 wound tissues of the HPL sheet treatment group, followed by less expression of fibrosis markers on day 28. These findings reflected the restored balance of the immune microenvironment stroke by ASC sheet application. Zimmermann et al. reported that MSCs modulate complex immune responses through paracrine effects, which aligns with our previous findings showing that HPL-cultured sheets expressed significant upregulation of C1q/TNF-related protein-3 *(CTRP3*) for immunomodulation. The regulation inhibited the C-C motif ligand 2 release by macrophages and subsequently reduced the chemotaxis of unstimulated macrophages [23, 36]. Furthermore, transplanted ASCs can act as a modulator, promptly dealing with the host’s immune responses and orchestrating the milieu composition by direct or indirect manners [37].

As for the regenerative aspect, several studies have revealed the efficacy of ASC transplantation at enhancing regeneration and angiogenesis in ischemic tissues [4, 19]. Kim et al. demonstrated the wound healing effect of ASCs was mainly facilitated by the production of angiogenic factors for angiogenesis [38]. In general, ASCs appeared to exhibit a better pro-angiogenic profile than BM-MSCs and umbilical cord-derived MSCs, as a large amount of angiogenic factors was detected in conditioned medium harvested from ASCs [39]. For example, ASCs can release HGF, which has been well-recognized for its potent pro-angiogenic properties [40]. HGF not only can promote formation of new vessels in repair process through stimulating vascular smooth muscle cells and endothelial cells [41], the association between abundant HGF expression in HPL sheet and the anti-fibrotic effect during wound healing process had also been revealed [23].

While the cell sheets possess multiple advantages, a decrease in VEGF expression has been observed upon sheet formation [23]. Some reports indicated that VEGF plays a diverse role in ASC-mediated regeneration via paracrine or autocrine mechanism [42]. Therefore, a reduction in VEGF secretion may hamper the regenerative effects of ASC sheets for ischemic tissues. In previous studies, ASC sheets were sandwiched by FGF2-tethered decellularized dermal matrix or transfected with VEGF to improve their potential in promoting angiogenesis [43, 44]. Nevertheless, the adoption of exogenous biomaterials or genetic engineering may contribute to unexpected adverse effects. Hence, it is imperative to propose a solution with higher biosafety assurance for this issue; for instance, Yu et al. enhanced the pro-angiogenic capability of ASC sheets by integrating the spheroids from the same source onto them [45]. In this study, the use of HPL for ASC sheet fabrication resulted in boosting the secretion of other specific cytokines, such as CCL5, that may rescue this disadvantage without obvious biosafety concerns.

Angiogenin, also named as secreted ribonuclease 5, is a 14.1-kDa monomeric protein of which the physiological role during inflammation is wound repair, exhibiting microbicidal activity and conferring host immunity [46]. Angiogenin is a multi-functional protein which participates in regulating the hematopoietic regeneration, vascularization and blood vessel homeostasis [47, 48], thereby being one of major biomarkers for predicting vascular regenerative efficacy of MSCs in the treatment of ischemic diseases [49]. Angiogenin is deemed important in the proangiogenic effects of microvesicles derived from MSCs [50], and it also plays a critical role in regulating angiogenesis and follicle survival when MSCs are co-transplanted with human ovarian tissues [51]. However, our data revealed that only relatively high concentration of angiogenin induced the formation of tube-like structures and proliferative activity of HUVECs. Hence, while angiogenin still contribute to the pro-angiogenic properties of HPL sheets, it may not be the main factor.

While CCL5 plays a pivotal role in the recruitment of various types of leukocytes and mediators into inflammatory sites [17], extensive pre-clinical in vitro and in vivo studies have highlighted the fundamental importance of the CCL5/CCR5 axis involved in VEGF-dependent angiogenesis in tumor microenvironment [52]. Similarly, the CCL5/CCR5 axis is intricately involved in the recruitment of endothelial progenitor cells (EPC) to the wound sites, facilitating neovascularization in tissue repair in a murine model. These EPCs were the source of growth factors that promoted angiogenesis as well [53]. Moreover, Kim et al. found that homing of skin-derived stem cells was mediated by CCL5 in a dose-dependent manner [54], which may bring synergistic effects to foster wound healing. Among the numerous paracrine factors released by ASCs to facilitate angiogenesis, the contribution of CCL5 has been noted previously [19]. Kimura et al. transplanted ASCs with reduced CCL5 expression to a bone fracture mouse model, resulting in unsatisfactory recovery of blood flow compared to the control [19]. Additionally, CCL5 signaling through CCR1 has been shown to regulate the stemness of ASCs, with the secretion of CCL5 by ASCs bolstering the repair of ischemic tissues [55]. Our study further elucidated the in vitro contribution of CCL5-related angiogenesis exerted by HPL sheets. Although the role of CCL5 was not further validated in vivo, our findings are valuable for future translational studies aiming to apply HPL-cultured ASC sheets in treating ischemic tissues or healing-impaired wounds.

## Conclusions

In this study, we demonstrated the efficacy of HPL-cultured ASC sheets in promoting wound healing using a burn injury model in rats. Following treatment with HPL sheets, the wound tissues exhibited enhanced angiogenesis and ASC engraftment. Additionally, the wound tissues treated with HPL sheets showed abundant collagen deposition with less fibrosis after healing, which could be attributed to reduced macrophage infiltration at the early stage of wound repair. Subsequent in vitro angiogenesis assays suggested that CCL5 released from HPL sheets played an important role in facilitating tube formation of endothelial cells. This regulatory mechanism was further confirmed through the application of CCL5 neutralizing antibody. Our data provided crucial evidence to suggest the involvement of CCL5 in HPL sheet-mediated angiogenesis, underscoring the potential clinical application of HPL sheets in wound healing. In the future, pre-clinical studies involving large animals are required for fully exploring the therapeutic potential of HPL-cultured ASC sheets.

### Electronic supplementary material

Below is the link to the electronic supplementary material.

Supplementary Material 1

## Data Availability

The raw microarray data were deposited in the Gene Expression Omnibus (GEO) under accession number GSE252798. The data can be accessed through the following link- https://www.ncbi.nlm.nih.gov/geo/query/acc.cgi?acc=GSE252798.

## Abbreviations

ASC: Adipose-derived stem cells
MSC: Mesenchymal stem cells
A2-P: Ascorbic acid 2-phosphate
ECM: Extracellular matrix
HGF: Hepatocyte growth factor
HPL: Human platelet lysate
FBS: Fetal bovine serum
FGF: Fibroblast growth factor
α-SMA: Alpha-smooth muscle actin
TGF-β1: Transforming growth factor-beta 1
HNA: Human nuclei antigen
ELISA: Enzyme-linked immunosorbent assay
CCL5: C–C motif chemokine ligand 5
HUVEC: Human umbilical vein endothelial cell
EBM: Endothelial basal medium
EGM: Endothelial growth medium
CM: Conditioned medium/media
CTRP3: C1q/TNF-related protein-3
BM: Bone marrow
VEGF: Vascular endothelial growth factor
