# Supplementary material for: Human platelet lysate-cultured adipose-derived stem cell sheets promote angiogenesis and accelerate wound healing via CCL5 modulation
Source: Stem Cell Res Ther. 2024 Jun 9;15:163. doi: 10.1186/s13287-024-03762-9 (PMC11163789; doi:10.1186/s13287-024-03762-9)
Supplement: Supplementary file 1 — Supplementary Material 1 [file 13287_2024_3762_MOESM1_ESM.docx]

**Supplementary Material:**

**Table S1.** Wound healing index of brown discoloration and scabbing/hardness

| Wound Healing Index | Brown Discoloration | Scabbing/Hardness |
| --- | --- | --- |
| 0 | no discoloration | normal skin |
| 1 | slight tan color | slight roughness and hardening edges not raised |
| 2 | light brown in color | moderate roughness and hardening slightly raised edges |
| 3 | moderate brown in color | hard rough scab and hardening moderately raised edges |
| 4 | maximum discoloration | hard rough scab |

**Table S2.** Primer sequences for quantitative PCR

| Gene | Forward (5' to 3') | Reverse (5' to 3') |
| --- | --- | --- |
| *CCL5* | CCTGCTGCTTTGCCTACATTGC | ACACACTTGGCGGTTCTTTCGG |
| *Angiogenin* | CAAGTCTTCTTTCCAGGTCAC | GGACAGGTAAGCCATTTTCAC |
| *GAPDH* | CAAGGCTGAGAACGGGAAGC | AGGGGGCAGAGATGATGACC |

**
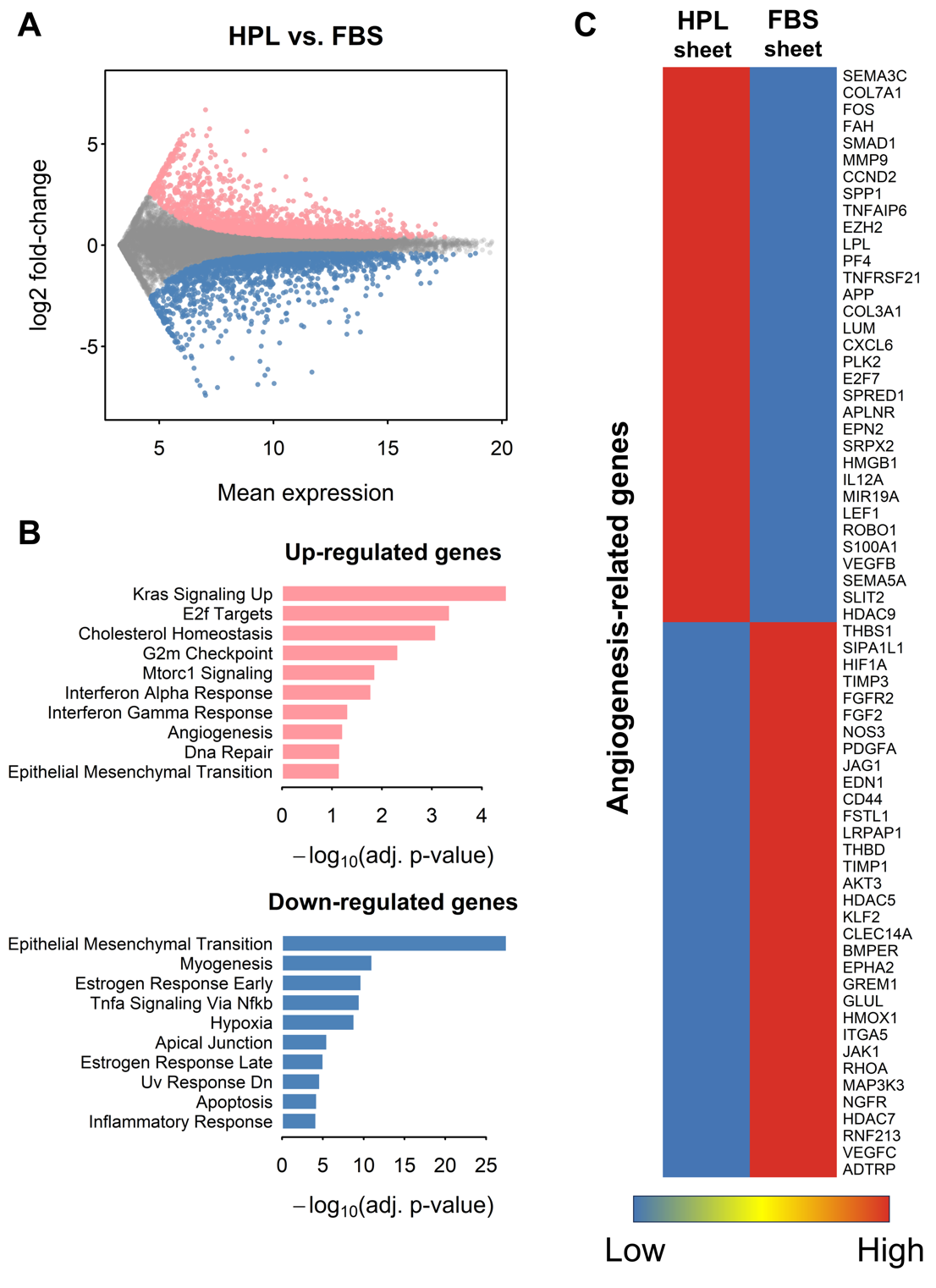
**

**Figure S1.** Angiogenesis-related genes induced in HPL-cultured ASC sheets. (A) The scatter plot illustrated the relationship between expression (x-axis) and log2 fold-change (y-axis) for each gene in response to FBS-cultured sheet compared to HPL-cultured sheet. Significantly up-regulated genes are highlighted in red (indicating higher expression in HPL sheet), while down-regulated genes are marked in blue (indicating higher expression in FBS sheet). (B) Pathway enrichment analysis showcasing the top 10 hallmark gene-sets from MSigDB significantly enriched in up-regulated (top) or down-regulated (bottom) genes. (C) The heatmap represented the expression patterns of angiogenesis-related genes between the HPL sheet and FBS sheet.

**
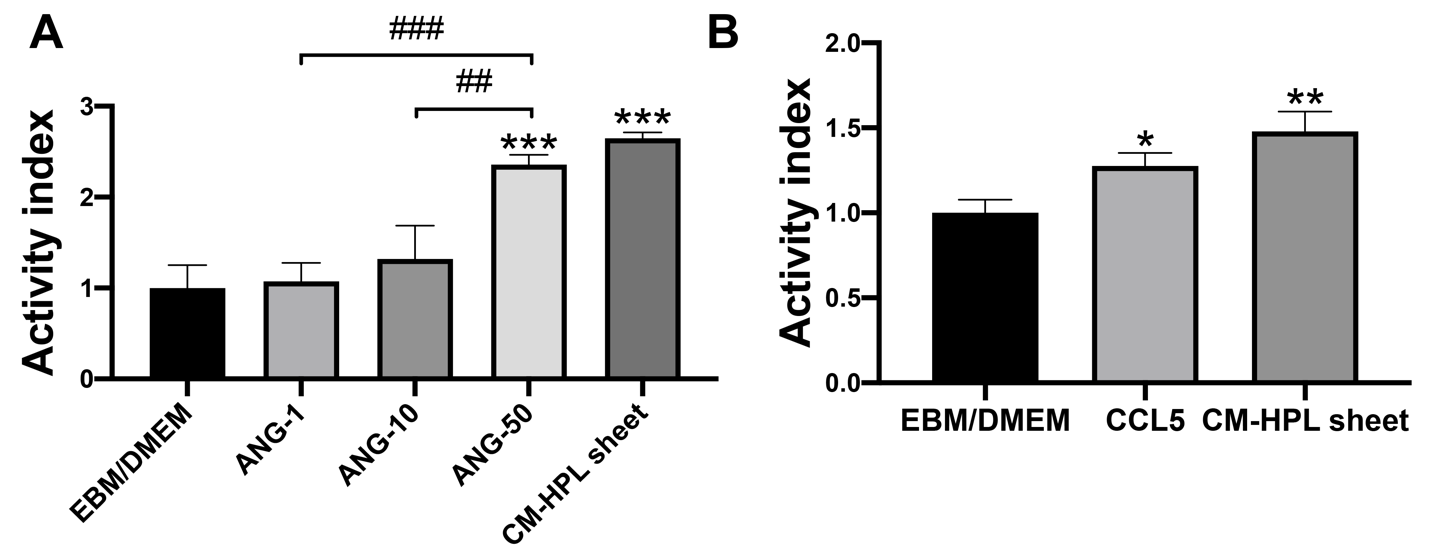
Figure S2.** Proliferative effect of exogenous CCL5 and angiogenin on endothelial cells

(A) Proliferative effect of CM from HPL sheets and certain concentrations of angiogenin was estimated (B) Proliferative effect of CM from HPL sheets and low concentration of CCL5 (1 ng/mL) was estimated. The activity index of ASCs was defined as the proliferative rate of each group relative to the control. **p* < 0.05, ***p* < 0.01, ****p* < 0.001 from the control (EBM/DMEM); ##*p* < 0.01, ###*p* < 0.001 between the indicated groups.
